# [3 + n] Cycloaddition Reactions: A Milestone Approach for Elaborating Pyridazine of Potential Interest in Medicinal Chemistry and Optoelectronics

**DOI:** 10.3390/molecules26113359

**Published:** 2021-06-02

**Authors:** Dorina Amariucai-Mantu, Violeta Mangalagiu, Ionel I. Mangalagiu

**Affiliations:** 1 Faculty of Chemistry, Alexandru Ioan Cuza University of Iasi, 11 Carol 1st Bvd, 700506 Iasi, Romania; dorina.mantu@uaic.ro; 2 CERNESIM Centre, Institute of Interdisciplinary Research, Alexandru Ioan Cuza University of Iasi, 11 Carol 1st Bvd, 700506 Iasi, Romania

**Keywords:** [3 + n] cycloaddition reactions, ylides, pyridazine, stereochemistry, regiochemistry, medicinal chemistry, optoelectronics

## Abstract

During the last few decades, pyridazine derivatives have emerged as privileged structures in heterocyclic chemistry, both because of their excellent chemistry and because of their potential applications in medicinal chemistry and optoelectronics. This review is focused on the recent advances in [3 + n] cycloaddition reactions in the pyridazine series as well as their medicinal chemistry and optoelectronic applications over the last ten years. The stereochemistry and regiochemistry of the cycloaddition reactions are discussed. Applications in optoelectronics (in particular, as fluorescent materials and sensors) and medicinal chemistry (in particular, antimicrobials and anticancer) are also reviewed.

## 1. Introduction

[3 + n] cycloaddition reactions represent one of the most important and efficient tools in the synthesis of heterocyclic rings, consisting of the addition of a multiple bond dipolarophile to a three-atom component system (TACS). Because of their complexity, [3 + 2] cycloaddition reactions have been extensively studied in the last 60 years [1,2,3,4,5,6,7,8,9,10,11,12,13,14], with interesting and heated discussions being involved in order to explain them.

**Scheme 1:**
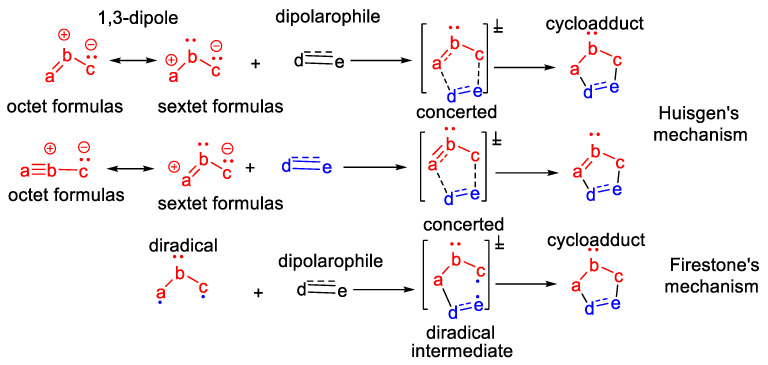
The [3 + 2] cycloadditions approach for obtaining five-membered heterocycles: Huisgen dipolar mechanism and Firestone diradical mechanism.

The [3 + 2] dipolar cycloaddition reactions were discovered by Rolf Huisgen in the early 1960s, more than 60 years ago [1,2]. The Huisgen [3 + 2] dipolar cycloaddition reactions represent an addition of a 1,3-dipole to a dipolarophile, leading to a five-membered heterocyclic ring [1,2,3,4,5,6,7], via a concerted mechanism, as presented in Scheme 1. The 1,3-dipole is a molecule with three atoms (abc), with a sextet of electrons (consequently, with a positive charge) to a marginal atom, and to the other marginal atom, a pair of non-bonding electrons (consequently, with a negative charge). The sextet formulas of the 1,3-dipole are stabilized by the non-bonding electrons of the central atom, adopting another canonical structure—the octet zwitterionic form. The dipolarophile is represented by an activated alkene or alkyne.

At the end of the 1960s, Firestone [9,10] proposed an alternative explanation for [3 + 2] cycloaddition reactions, via a radical mechanism (Scheme 1). In his approach, Firestone proposed a two-step mechanism, via the formation of a diradical intermediate.

Over the last decade, Domingo’s team [11,12,13,14] have revealed new, interesting considerations concerning the mechanistic inside of [3 + 2] cycloaddition reactions, based on molecular electron density theory (MEDT). Using their research, they classify the TACS in pseudo-diradical, pseudoradical, zwitterionic and carbenoid. Using MEDT theory, [3 + 2] cycloaddition reactions have been classified by Domingo into the corresponding pseudo-diradical, pseudoradical, zwitterionic and carbenoid reactions [11,12,13,14].

**Scheme 2:**
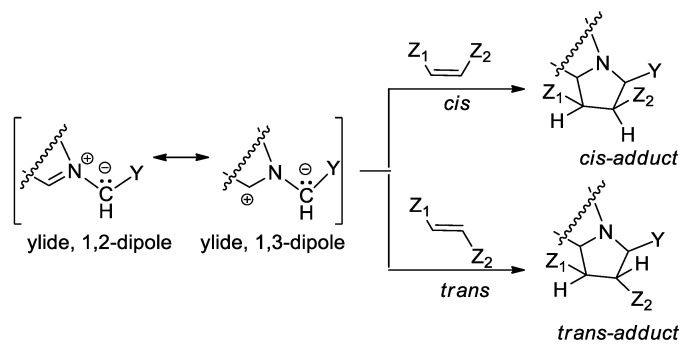
Stereochemistry of addition of cycloimmonium ylides to symmetrically substituted *cis* and *trans* olefins.

An interesting case of [3 + 2] cycloaddition reactions is represented by the cycloaddition of cycloimmonium ylides to various symmetrically and non-symmetrically substituted dipolarophiles, Scheme 2. The [3 + 2] cycloaddition reactions of cycloimmonium ylides to symmetrically substituted *Z* (or *cis*) and *E* (or *trans*) olefins involve interesting problems of stereochemistry, these reactions being highly stereospecific [8,15,16].

**Scheme 3:**
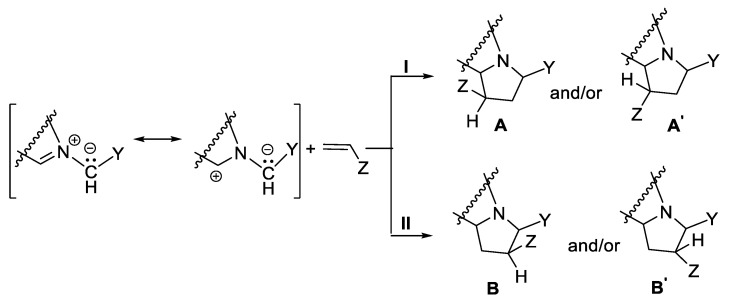
Regiochemistry of addition of cycloimmonium ylides to non-symmetrically substituted dipolarophiles, with the formation of endo/exo **A** and **A’**, and **B** and **B’** pairs of stereoisomers.

The [3 + 2] cycloaddition reactions of cycloimmonium ylides to non-symmetrically substituted dipolarophiles (with double or triple bond) involve interesting combined stereo- and regiochemistry aspects [17,18,19,20,21]. In the regiochemistry case, because of the double sense of the addition of ylide to dipolarophile, two reactions pathways (I or II) are possible, with the formation of two pairs of regioisomers (**A**, **A’** and **B**, **B’**, Scheme 3), according to orbital, steric and electronic factors.

On the other hand, [3 + 2] cycloaddition reactions represent an accessible and facile tool to obtain pyridazine derivatives of potential interest in medicinal chemistry (as antibacterials, antifungals, antituberculars, antivirals, anticancer agents, anti-inflammatories, antihypertensives, diuretics, etc.) [22,23] and in optoelectronics (as highly fluorescent materials, sensors and biosensors, lasers, etc.) [24].

From a previous review conducted ten years ago [23], it is clear that the focus has been on the most important achievements in the chemistry and applications of 1,2-diazine (pyridazine and phthalazine). In the present review, we focused on the recent advances in [3 + n] cycloaddition reactions in the pyridazine series as well as their medicinal chemistry and optoelectronic applications in the last ten years.

## 2. Results and Discussions

### 2.1. [3 + 2] Cycloaddition Reactions in the Pyridazinium and Phthalazinium Ylides Series

**Scheme 4:**
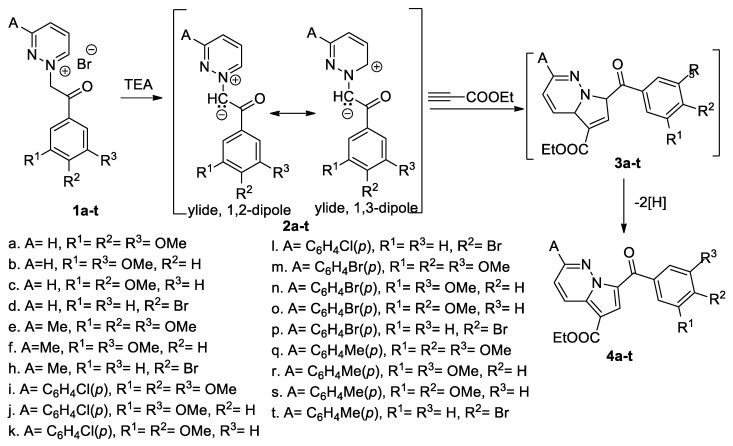
The [3 + 2] cycloaddition of pyrrolo[1,2-b]pyridazine derivatives **4a**–**t**.

Popovici et al. [25] synthesized different pyrrolo [1,2-b]pyridazine derivatives (**4a**–**t**), using the [3 + 2] cycloaddition reactions of different pyridazinium ylides (**2a**–**t**; generated in situ from the corresponding pyridazinium salts (**1a**–**t**), in the presence of triethylamine (TEA) as the base), to ethyl propiolate, as shown in Scheme 4.

In the first instance, the corresponding intermediate dihydropyrrolo[1,2-b]pyridazines **3a**–**t** were obtained and underwent an oxidative dehydrogenation under atmospheric conditions, leading to the final pyrrolo[1,2-b]pyridazine derivatives **4a**–**t**, in moderate yields (40–52%). The [3 + 2] cycloadditions occur completely regioselectively, with a single type of regioisomer (**4**) being obtained. Some of the obtained pyrrolo[2,1-b]pyridazines were tested in vitro against a panel of 60 human tumor cell lines at a single dose up to five doses (according to National Cancer Institute protocol). The adducts **4a**, **4b**, **4e**, and **4f** showed a very good growth inhibition effect on almost all 60 cell lines, the best results being registered on HL-60 (TB) leukemia cells, COLO205 colon cancer cells, MDA-MB-435 melanoma cells, OVCAR-3 ovarian cancer cells and A498 renal cancer cells. The most active compound was **4f** with GI_50_ values <100 nM on thirteen cell lines including colon, ovarian, renal, prostate, brain and breast cancer, melanoma and leukemia. Docking experiments have been performed for the biologically active cycloadducts (**4a**, **4b**, **4e**, **4f**) and showed good compatibility with the colchicine binding site of tubulin. The authors claim that cycloadduct **4f** could serve as a useful lead in anticancer drug design.

**Scheme 5:**
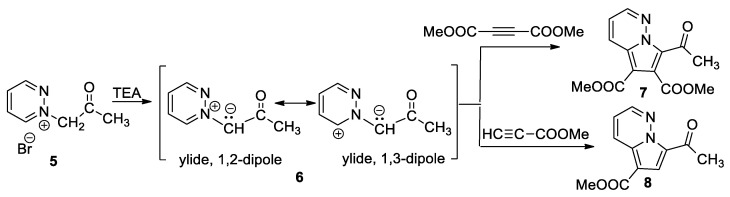
The [3 + 2] cycloaddition of pyridazinium ylides with DMAD and methyl propiolate.

In continuation of their concern for new fluorescent pyrrolodiazine derivatives, Moldoveanu et al. [26] performed an interesting study concerning the cycloaddition reactions of pyridazinium ylides **6** (generated in situ from the corresponding pyridazinium salts **5** in the presence of TEA) in activated symmetrically and non-symmetrically substituted dipolarophiles with a triple bond (dimethyl acetylenedicarboxylate–DMAD and methyl propiolate), as shown in Scheme 5.

A reaction with DMAD leads to the azabicycle **7**, while [3 + 2] cycloaddition of ylide with methyl propiolate occurs completely regioselectively, with a single regioisomer (**8**) being obtained. The reactions were performed both under conventional thermal heating (TH) as well as under unconventional heating (microwave (MW) irradiation). The authors claim that under MW irradiation, the yields are higher, at around 10–15%. The authors prove that the cycloadducts **7** and **8** are intense blue emitters and have a quantum yield around 25%.

**Scheme 6:**
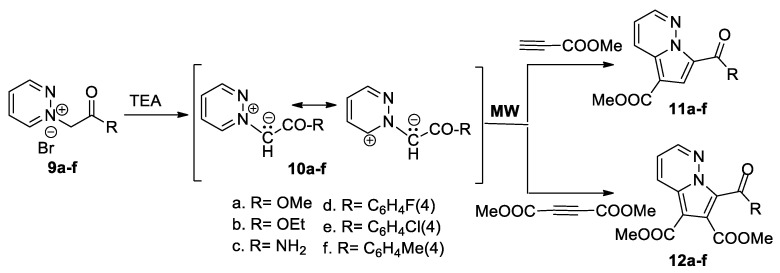
The [3 + 2] cycloaddition of pyridazinium ylides **10a**–**f** with DMAD and methyl propiolate.

In continuation of their work in the field of cycloaddition reactions, Zbancioc et al. [27,28] reported an interesting and detailed study concerning the thermal versus specific effects of MW in the [3 + 2] cycloaddition reactions. In this respect, they studied the [3 + 2] cycloaddition reactions of pyridazinium ylides **10a**–**f** (generated in situ from the corresponding pyridazinium salts **9a**–**f** in the presence of TEA) to methyl propiolate and DMAD, as shown in Scheme 6.

The cycloaddition reactions of pyridazinium ylides **10a**–**f** to methyl propiolate occur completely regioselectively, with a single type of regioisomer being obtained, namely pyrrolopyridazine **11a**–**f**. The reaction with DMAD leads to the pyrrolopyridazines **12a**–**f**. The reactions were studied under MW irradiation, in order to elucidate the intrinsic mechanism of MW effects. On the basis of their study, the authors conclude that there are no specific MW effects in the studied [3 + 2] cycloaddition reactions, the observed effects being purely thermal. Time-dependent density functional theory computations and steady-state electronic spectroscopy measurements were performed for the pyrrolopyridazine **11a** and **11e**, revealing that these compounds possess an intense blue fluorescence [28].

**Scheme 7:**
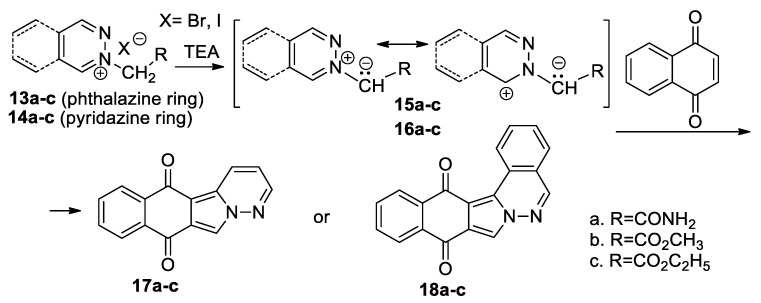
The [3 + 2] cycloaddition of pyridazinium and phthalazinium ylides to 1,4-naphthoquinone.

Mantu et al. [29,30,31] performed a straightforward and efficient study concerning the [3 + 2] cycloaddition reactions of pyridazinium **15a**–**c** and phthalazinium **16a**–**c** ylides (generated in situ from the corresponding pyridazinium **13a**–**c** and phthalazinium **14a**–**c** salts in the presence of TEA), to 1,4-naphthoquinone, as symmetrically activated cyclic alkene (Scheme 7).

The reaction leads either to polycyclic dihydrobenzo[f]pyridazino[6,1-a] isoindole **17a**–**c** or to dihydrobenzo[5,6]isoindolo[1,2-a]phthalazine **18a**–**c**. The reactions were performed under conventional TH, as well as under MW or ultrasound (US) irradiation. The obtained data show that MW and US irradiation produce a remarkable acceleration for reactions (from hours to minutes), the consumed energy decreases considerably and the yields are higher. The US irradiation was found to be the most effective method, in terms of yields and reaction time, for obtaining polycyclic 1,2-diazines. The in vitro anticancer assay [30] against an NCI 60 human tumor cell line panel reveals that the polycyclic compounds **17a**–**c** and **18a**–**c** have anticancer activity. The obtained data led the authors to claim that polycyclic compounds **17a**–**c** and **18a**–**c** will act as an anticancer vector through multiple mechanisms of action, the intercalation with the DNA prevailing in competition with the other mechanisms.

**Scheme 8:**
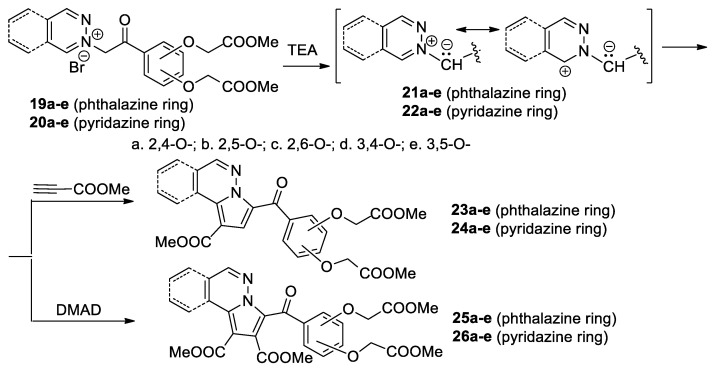
The [3 + 2] cycloaddition of pyridazinium and phthalazinium ylides with dihydroxyacetophenone skeleton.

Zbancioc et al. [32,33,34] performed a thorough study concerning the [3 + 2] cycloaddition reactions of pyridazinium **21a**–**e** and phthalazinium **22a**–**e** ylides with the dihydroxyacetophenone skeleton (generated in situ from the corresponding pyridazinium **19a**–**e** and phthalazinium **20a**–**c** salts in the presence of TEA), to methyl propiolate and DMAD, as shown in Scheme 8.

The [3 + 2] cycloaddition reaction of the ylides **21a**–**e** and **22a**–**e** with methyl propiolate occur completely regioselectively, with a single type of regioisomer being obtained—the fused pyrrolo-diazines with the dihydroxyacetophenone skeleton **23a**–**e** and **24a**–**e**. In the case of the cycloaddition reaction of ylides **21a**–**e** and **22a**–**e** with the symmetrically substituted alkynes DMAD, the aromatized fused pyrrolo-diazines with the dihydroxyacetophenone skeleton **25a**–**e** and **26a**–**e** are obtained. The reactions were performed under conventional TH, as well as under MW or US irradiation. Under MW and US irradiation, the yields are higher, the reaction time decreases substantially (from hours to minutes), the consumed energy decreases considerably, the amount of used solvent also decreases, and the reaction conditions are milder. As a result, these reactions could be considered environmentally friendly. Overall, the use of US proved to be more efficient than MW or TH. The in vitro anticancer assay proves that some of the obtained compounds have a significant antitumor activity and a moderate antifungal activity, while the antibacterial activity is negligible [34].

**Scheme 9:**
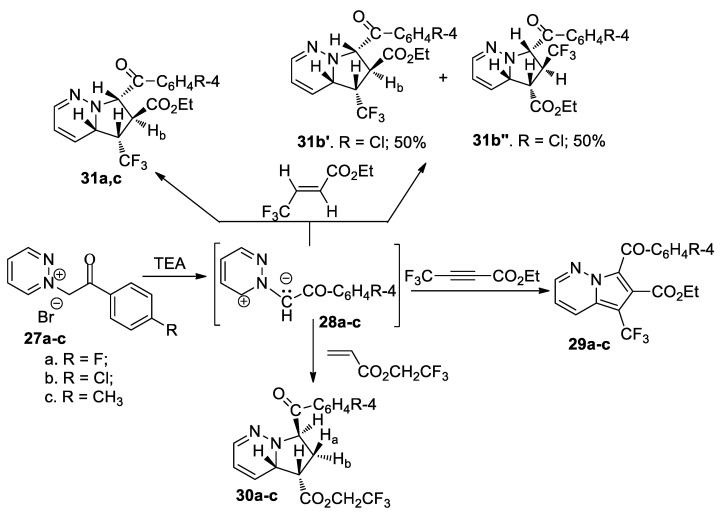
The [3 + 2] cycloaddition of pyridazinium ylides **28a**–**c** to various fluorine dipolarophiles with double and triple bond.

Tucaliuc et al. [35] performed an interesting study concerning the [3 + 2] cycloaddition reactions of pyridazinium ylides **28a**–**c** with various activated non-symmetrically substituted dipolarophiles (alkenes and alkynes) containing fluorine moiety, as shown in Scheme 9.

The [3 + 2] cycloaddition reaction of ylides **28a**–**c** to 2,2,2-trifluoroethyl acrylate occur completely regioselectively, involving a single type of regioisomer being obtained, with a tetrahydropyrrolopyridazine structure **30a**–**c**. The cycloaddition reaction of ylides **28a**–**c** to ethyl 4,4,4-trifluorobutinoate leads to the aromatized pyrrolopyridazine derivatives **29a**–**c**, again with the reaction being completely regioselective.

When the dipolarophile was ethyl 4,4,4-trifluorocrotonate (*E*-isomer), the reactions involved additional stereo- and regiochemical problems. The cycloaddition reaction of ylides **28a** and **28c** occurs completely stereo- and regioselectively, with only isomers **31a** and **31c** being obtained. In the case of ylide **28b** (R=Cl), the cycloaddition reaction occurs regioselectively, with two regisomers **31b′** and **31b’’** being obtained, in a molar ratio of 1:1.

These reactions were performed under conventional TH and under MW or US irradiation (both in liquid phase and phase-transfer catalysis). The use of MW irradiation proved to be more efficient (with the reaction time decreasing substantially, higher yields, smaller amount of used solvent), with these reactions being considered environmentally friendly. The in vitro antimicrobial activity of the obtained compounds indicated that the introduction of a trifluoromethyl moiety on the pyridazine skeleton is beneficial for antimicrobial activity. Practically all compounds have a spectacular antimicrobial activity against Gram positive germs, and very good activity against Gram negative germs. The antifungal activity of compounds was negligible.

**Scheme 10:**
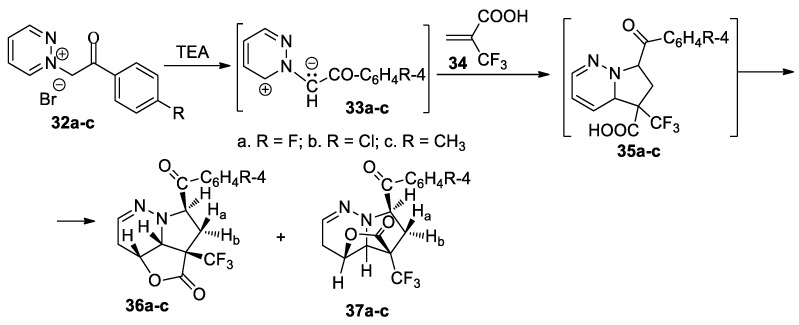
The [3 + 2] cycloaddition of pyridazinium ylides **33a**–**c** to 2-(trifluoromethyl)acrylic acid.

Butnariu et al. [36] performed a straightforward, efficient and selective study for obtaining hybrid trifluoromethyl-substituted γ-lactones with nitrogen heterocyclic skeletons **36a**–**c** and **37a**–**c**, as shown in Scheme 10.

The [3 + 2] cycloaddition reaction of ylides **33a**–**c** to 2-(trifluoromethyl)acrylic acid **34** occurs unexpectedly; instead of the cycloadducts **35a**–**c**, a new class of organic compounds were obtained: fused γ-lactones with nitrogen heterocyclic skeletons—**36a**–**c** and **37a**–**c**. As for regiochemistry, the cycloaddition reactions occur regioselectively, with the formation of one pair of stereoisomers (type **A** and **A’**, Scheme 3), namely **36a**–**c** (major) and **37a**–**c**. A feasible reaction mechanism is presented for γ-lactone formation via a cascade reaction: an initial [3 + 2] cycloaddition leads to intermediary cycloadducts **35a**–**c**, which underwent a concomitant protonation of the nitrogen atom and nucleophilic attack of the carboxylate oxygen at the adjacent endocyclic carbocation.

### 2.2. [3 + n] Cycloaddition Reactions in the Nitrile Imine series

**Scheme 11:**
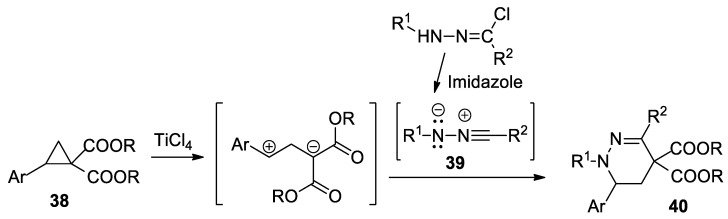
The [3 + 3] cycloaddition reactions of nitrile imines to donor–acceptor cyclopropanes.

Grave et al. [37], using [3 + 3] cycloaddition reactions of various nitrile imines **39** to donor–acceptor cyclopropanes **28**, report a straightforward and efficient synthesis of tetrahydropyridazine derivatives **40** (23 products), as shown in Scheme 11.

The donor–acceptor cyclopropanes (Ar are various aromatic radicals: phenyl, 2-/3-/4-substituted-phenyl) were activated by a catalytic amount of TiCl_4_. The nitrile imines (R^1^ and R^2^ are different radicals—phenyl, substituted phenyl, naphthyl, benzoyl, thienyl) were generated in situ from hydrazonyl chlorides by treatment with imidazole.

**Scheme 12:**
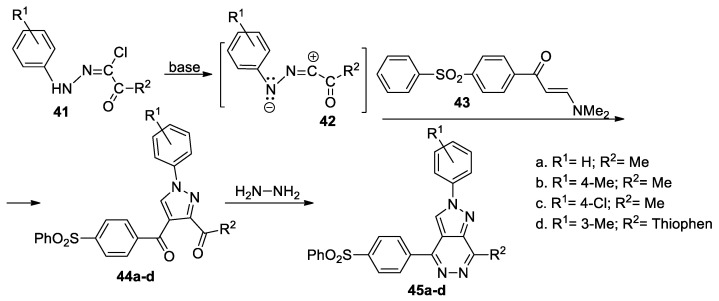
Two-step synthesis of pyrazolo[3,4-d]pyridazines **45a**–**d**.

Mady et al. [38] report a green, facile and efficient synthetic approach for pyrazolo[3,4-d]pyridazines linked to sulfone moiety **45a**–**d**, as shown in Scheme 12. In order to obtain the desired compounds **45a**–**d**, initially, they obtain the pyrazole derivatives **44a**–**d**, using the cycloaddition reaction of the enaminones **43** with the nitrile imines **42** (generated in situ by the action of the base on the hydrazonoyl chlorides **41**), followed by a cyclocondensation with hydrazine.

The reactions were performed using both CT heating and MW irradiation, with the reactions under MW being more efficient in terms of yields and time. The pyrazolo[3,4-d]pyridazine derivatives **45a**–**d** were tested for their antimicrobial activity, the compounds having a very good antibacterial activity but not antifungal.

**Scheme 13:**
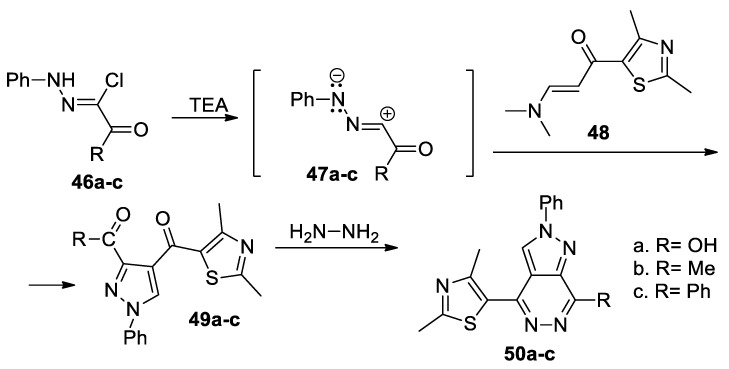
Two-step synthesis of pyrazolo[3,4-d]pyridazines **50a**–**c**.

Zaki et al. [39] synthesized a series of pyrazolo[3,4-d]pyridazines **50a**–**c**, as shown in Scheme 13. In order to obtain the desired compounds **50a**–**c**, initially they obtain the pyrazole derivatives **49a**–**c** using the cycloaddition reaction of the nitrile imines **47a**–**c** (generated in situ by the action of the base TEA on the hydrazonoyl chlorides **46a**–**c**) with thiazole derivative **48**, followed by a cyclocondensation with hydrazine.

The pyrazolo[3,4-d]pyridazine derivatives **50a**–**c** were tested for their antimicrobial activity, the compounds having a good antibacterial activity (both on Gram positive and Gram negative bacteria) but not antifungal.

**Scheme 14:**
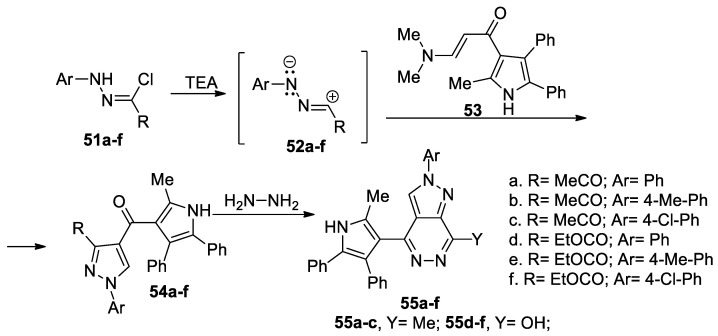
Two-step synthesis of pyrazolo-pyridazines **55a**–**f**.

Eldebss et al. [40] synthesized a series of pyrazolo-pyridazines **55a**–**f**, as shown in Scheme 14. In order to obtain the desired compounds **55a**–**f**, initially they obtain the pyrazole derivatives **54a**–**f**, using the cycloaddition reaction of the nitrile imines **52a**–**f** (generated in situ by the action of the base TEA on the hydrazonoyl chlorides **51a**–**f**) with enaminone-pyrrolo derivative **53**, followed by a cyclocondensation with hydrazine. The cycloaddition reaction occur regioselective, in good yields.

The pyrazolo-pyridazine derivatives **55a**–**f** were tested for their protein kinase inhibitory activities against 25 kinases (belonging to four kinase groups), with the obtained results revealing that the compounds are selective and very good inhibitors against VEGFR-2, EGFR and CHK1, with IC_50_ values in the sub-micromolar range.

**Scheme 15:**
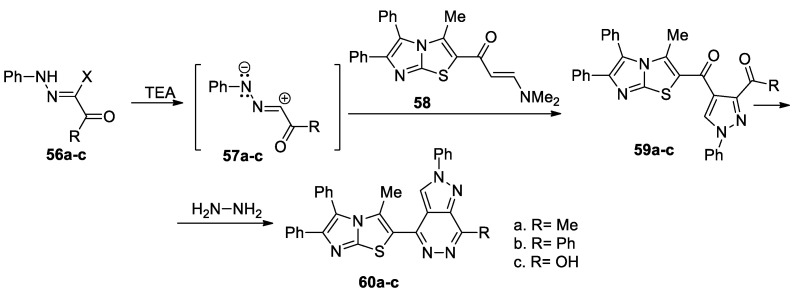
Two-step synthesis of pyrazolo[3,4-d]pyridazines **60a**–**c**.

Gomha et al. [41] report the synthesis of a large variety of pyrazolo-azaheterocycles of potential interest in medicinal chemistry, as shown in Scheme 15. In order to obtain the desired compounds **60a**–**c**, initially they obtain the pyrazole derivatives **59a**–**c**, using a regioselective cycloaddition of nitrile imines **57a**–**c** (generated in situ by the action of the base on the hydrazonoyl chlorides **56a**–**c**) with enaminone **58**, followed by a cyclocondensation with hydrazine hydrate when the corresponding pyrazolo[3,4-d]pyridazines **60a**–**c** are obtained.

**Scheme 16:**
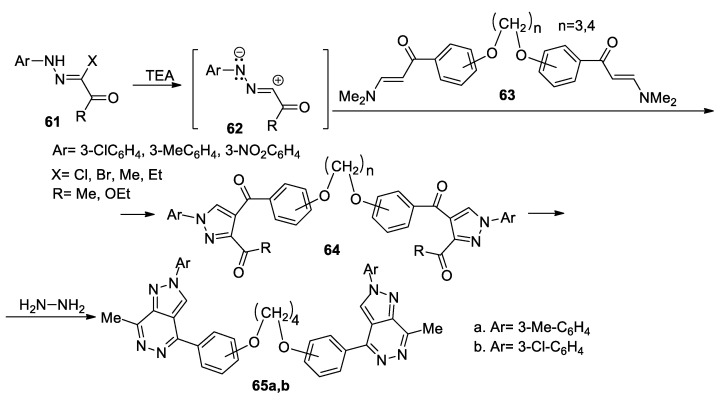
Two-step synthesis of *bis*-pyrazolo[3,4-d]pyridazines **65a**,**b**.

Using a similar strategy, Elwahy et al. [42] report an interesting study concerning synthesis of *bis*-pyrazolo[3,4-d]pyridazines **65a**,**b**, as shown in Scheme 16. In order to obtain the desired compounds **65a**,**b**, initially they obtain the *bis*-pyrazole derivatives **64**, using the cycloaddition reactions of nitrile imines **62** (generated in situ by the action of the base on the hydrazonoyl chlorides **61**) with *bis*(enaminones) **63**, followed by a cyclocondensation with hydrazine hydrate when the corresponding *bis*-pyrazolo[3,4-d]pyridazines **65a,b** are obtained.

These reactions were performed under conventional TH and MW irradiation. The author claims that MW irradiation is beneficial for the reaction pathway, the reaction conditions are milder, the yields are higher and the reaction time decreases.

**Scheme 17:**
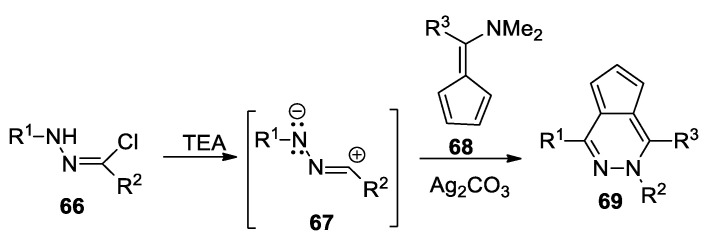
Synthesis of cyclopenta[d]pyridazines **69** by cycloaddition reactions.

Abranyi-Balogh et al. [43] report a convenient and direct one-pot synthesis of cyclopenta[d]pyridazines, as shown in Scheme 17. In order to obtain the desired compound **69**, they use the cycloaddition reactions of nitrile imine **67** (generated in situ from the hydrazonoyl chlorides **66**; R^1^ = methyl, iso-propyl, tert-butyl, cyclohexyl, 3-OMe-phenyl; R^2^ = 4-OMe-phenyl) with fulvene **68** (R^3^ = H, methyl), using Ag_2_CO_3_ as the catalyst.

These reactions were performed under mild conditions and the yields were good to high.

### 2.3. Pyridazine Derivatives Obtained by Cycloaddition and/or by Click Reactions of Azomethine Ylides or Imines

**Scheme 18:**
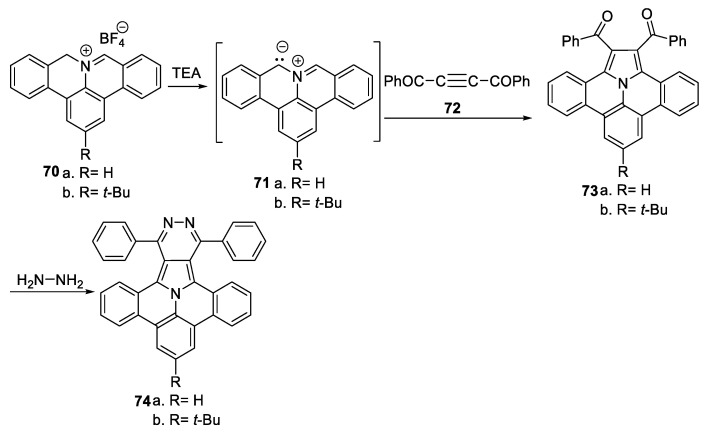
Two-step synthesis of polycyclic aromatic compounds with pyrrolopyridazine core **74a**,**b**.

Richter et al. [44,45] performed an in-depth study concerning the optoelectronic properties of and obtaining some polycyclic aromatic hydrocarbons with pyrrolopyridazine core **74a**,**b**, as shown in Scheme 18.

The synthesis was performed in two steps: an initial cycloaddition reaction of azomethine ylides **71a**,**b** (generated from the salts **70a**,**b** and TEA) to symmetrically substituted dipolarophiles with a triple bond (namely, 1,4-diphenylbut-2-yne-1,4-dione **72**) leads to the intermediary ullazine **73a**,**b**; this reaction is followed by a cyclocondensation of **73a**,**b** with hydrazine, when the desired products **74a**,**b** are obtained. In the obtained polycyclic aromatic hydrocarbons with pyrrolopyridazine core **74**, interesting intramolecular push–pull phenomena between the ullazine part (as donor) and the pyridazine core (as acceptor part) were observed, which makes these compounds suitable candidates for organic field effect transistor (OFET) applications or chemical/bio-sensing. Similar results were obtained in this group (Berger) for other azaheterocycles [45].

**Scheme 19:**
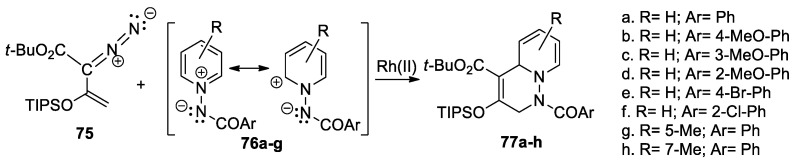
Synthesis of tetrahydropyridazine derivatives **80a**–**h** by cycloaddition reactions.

Xu et al. [46,47] synthesized a series of substituted 1,2,3,6-tetrahydropyridazine derivatives **77a**–**h**, using the dirhodium-catalyzed [3 + 3] cycloaddition reactions of different *N*-acylimino-pyridinium ylides **76a**–**g**, to enol diazoacetates **75** [TIPS = *tri*isopropylsilyl], as shown in Scheme 19.

The reactions occur regioselectively, with high yields, and excellent enantio-selectivities, controlled by the reaction conditions and catalysts. The sequence of reactions is triggered by Rh(II)-catalyzed dinitrogen extrusion, followed by vinylogous addition with *N*-acyliminopyridinium ylides.

**Scheme 20:**
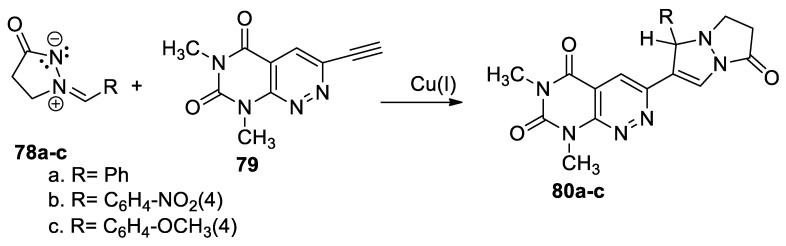
Copper-catalyzed [3 + 2] cycloaddition reactions for synthesis of **80a**–**c**.

Using the cycloaddition reactions of azometine imines to π-deficient alkynyl hetarenes (used as dipolarophiles), Nelina-Nemtseva et al. [48] obtained various classes of fused azaheterocycles, these including pyrimido[4,5-c]pyridazine derivatives **80a**–**c**, as shown in Scheme 20.

In order to obtain the fused pyridazine derivatives **80a**–**c**, the authors used the copper-catalyzed [3 + 2] cycloaddition reactions. In this respect, 2-arylidene-5-oxopyrazolidin-2-ium-1-ides **78a**–**c** were coupled with 3-ethynyl-6,8-dimethylpyrimido[4,5-c]pyridazine-5,7(6H,8H)-dione **79** (as dipolarophile), using Cu(I) as the catalyst. The reactions occur in moderate to excellent yields.

**Scheme 21:**
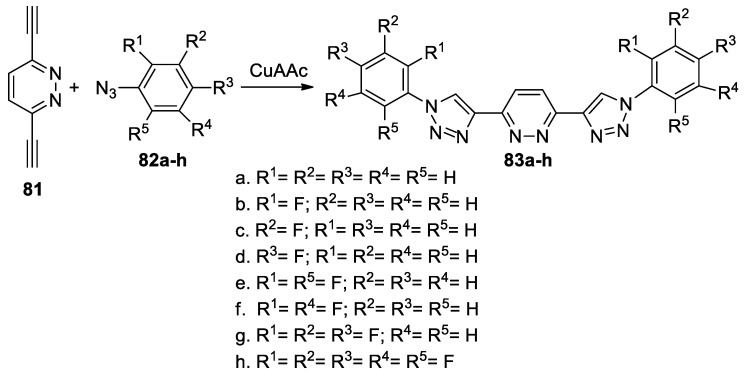
Copper-catalyzed [3 + 2] cycloaddition reactions for synthesis of **83a**–**h**.

Birkenfelder et al. [49] synthesized a series of 3,6-*bis*(4-triazolyl)pyridazines by using a facile copper-catalyzed [3 + 2] cycloaddition reaction, as shown in Scheme 21.

In this respect, 3,6-ethynylpyridazine **81** (used as dipolarophile with a triple bond) was treated with the corresponding azides **82a**–**h** (CuAAC was used as a catalyst for click reactions), leading to the desired 3,6-*bis*(4-triazolyl)pyridazines **83a**–**h**. Electrochemistry and optical spectroscopy of the obtained 3,6-*bis*(4-triazolyl)pyridazines **83a**–**h** suggest that these compounds have an *n*-type organic semiconductor behavior, which make them useful as electron-transporting/hole-blocking materials in optoelectronics.

**Scheme 22:**
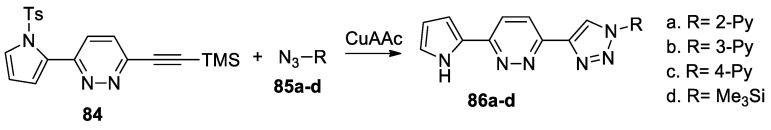
Azide-alkyne cycloaddition reactions for synthesis of pyridazine derivatives **86a**–**d**.

Yang et al. [50] synthesized a series of pyrrolo-pyridazyl-triazolyl derivatives **86a**–**d**, by using a facile copper-catalyzed azide-alkyne cycloaddition reaction, as shown in Scheme 22.

In this respect, pyrrolo-pyridazine derivative **84** (used as dipolarophile with triple bond) was treated with the corresponding azides **85a**–**d** (using CuAAC as catalyst for click reactions), leading to the desired pyrrolo-pyridazyl-triazolyl derivatives **86a**–**d**. The authors suggest that these compounds could have applications in optoelectronics as π-conjugated D1-A1-D2-A2 materials.

### 2.4. Miscellaneous [3 + n] Cycloaddition Reactions

**Scheme 23:**
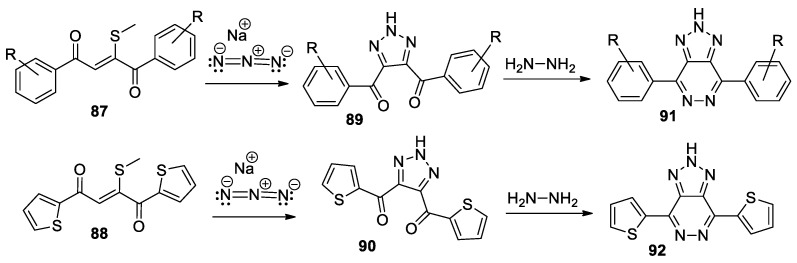
Two-step synthesis of 1,2,3-triazolo-pyridazine **91** and **92**.

Swarup et al. [51] synthesized a series of 1,2,3-triazolo-pyridazine derivatives **91** and **92**, using a straightforward and efficient procedure. In this respect, initially they obtain the 4,5-disubstituted-1,2,3-triazoles **89** and **90**, through the cycloaddition reactions of R-benzo- or 2-methylthio- 1,4-ene-dione (compounds **87** and **88)** and sodium azide (Scheme 23), followed by a cyclocondensation with hydrazine.

The triazolo-pyridazine derivatives, especially **92**, could be used as key starting materials for obtaining solar cells.

**Scheme 24:**
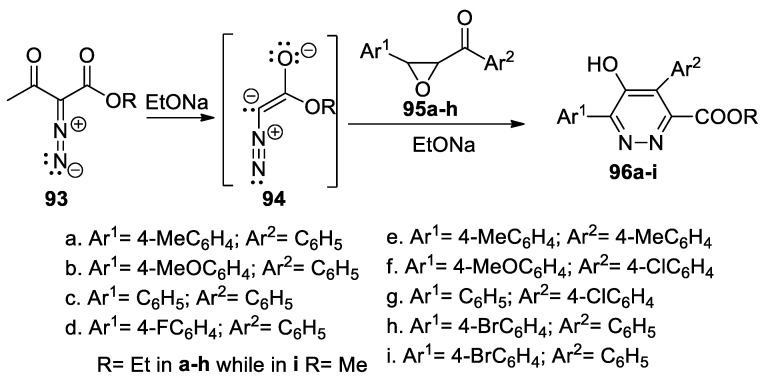
Synthesis of pyridazine esters **96a**–**i** by [3 + 3] annulation reactions.

Nair et al. [52] synthesized a series of highly functionalized pyridazine esters **96a**–**i** by cycloaddition reactions, as shown in Scheme 24. As 1,3-dipole, they used an α-diazoester anion **94** (generated in situ from the α-diazo-β-ketoester **93** in the presence of a base, EtONa), which undergoes a [3 + 3] annulation with chalcone epoxides **95a**–**h**.

The reactions occur in moderate yields but completely regioselectively, in mild conditions, and with a wide variety of functional groups.

**Scheme 25:**
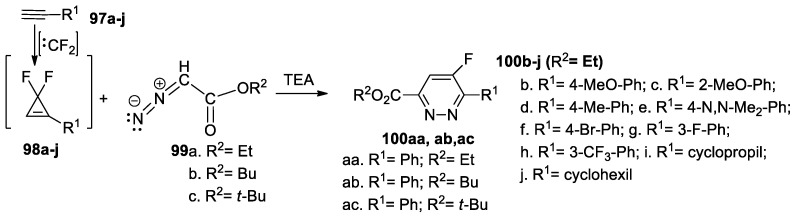
Synthesis of fluoro-pyridazine derivatives **100a**–**j** by [3 + 2] cycloaddition reactions.

Tran et al. [53] synthesized different fluoro-pyridazine derivatives **100a**–**j**, using the [3 + 2] cycloadditions of different R^2^-diazoacetate derivatives **99a**–**c**, in the presence of TEA, to different 1-R^1^-2,2-difluorocyclopropene **98a**–**j** (generated in situ from the corresponding acetylenic derivatives **97a**–**j** and difluorocarbene), as shown in Scheme 25.

Overall, the reaction pathway is direct and efficient, with the desired 5-fluoro-pyridazines **100a**–**j** being obtained in modest to good yields (30−86%).

**Scheme 26:**
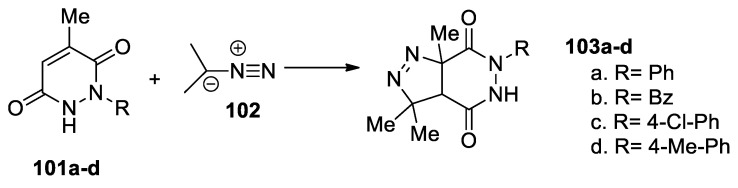
Synthesis of pyrazolo[3,4-d]pyridazinones **103a**–**d** by [3 + 2] cycloaddition reactions.

Ben Hamadi et al. [54] report an efficient and straightforward synthesis of saturated pyrazolo-pyridazinone via cycloaddition reactions, as shown in Scheme 26. In this respect, they use a [3 + 2] cycloaddition reaction of diazopropane **102** to pyridazinones **101a**–**d**, when the corresponding saturated pyrazolo[3,4-d]pyridazinones **103a**–**d** are obtained.

These reactions were performed under conventional TH and US irradiation, concluding that the use of US irradiation has substantial advantages, with yields being higher, the reaction time decreasing, and the toxicity decreasing by using nontoxic solvents.

## 3. Concluding Remarks

In conclusion, it is clear that the [3 + n] cycloaddition reactions in the pyridazine series remain a versatile and useful tool to obtain new compounds of potential interest in medicinal chemistry and optoelectronics. The theoretical and experimental aspects of stereochemistry and regiochemistry involved in the [3 + n] cycloadditions are complex and attractive, and continue to rouse the interest of scientific community.
